# Spectator to a Meeting of Minds: Vicarious Eye Contact Elicits Psychophysiological Responses in the Observer

**DOI:** 10.1111/psyp.70386

**Published:** 2026-08-24

**Authors:** Samuli Linnunsalo, Jari K. Hietanen

**Affiliations:** ^1^ Human Information Processing Laboratory, Faculty of Social Sciences/Psychology Tampere University Tampere Finland

## Abstract

Research has shown that dyadic eye contact elicits psychophysiological responses related to affect and attention. In daily life, we also frequently notice eye contact occurring between other people, even when we are not directly involved. This study investigated whether such *vicarious eye contact* elicits affect‐ and attention‐related psychophysiological responses in observers. In two experiments, we measured participants' skin conductance (affective arousal), facial muscle activity (affective valence/affiliation), and heart rate deceleration (attention orienting) responses while they viewed gaze interactions between two agents. In Experiment 1, participants observed eye contact between two humans, whereas in Experiment 2, participants saw a human and a humanoid robot NAO. We found novel evidence for a *vicarious eye contact effect*: viewing eye contact between two humans elicited greater affective arousal in observers compared to non‐eye contact conditions. Somewhat surprisingly, facial muscle responses indicated increased zygomaticus activity and decreased corrugator activity during vicarious eye contact relative to non‐eye contact conditions when participants viewed eye contact between a human and a humanoid robot, but not when viewing eye contact between two humans. In both experiments, vicarious eye contact failed to elicit a greater heart rate deceleration response, suggesting that it lacks the attention‐capturing characteristics of dyadic eye contact. We discuss these findings in the context of mind attribution in gaze interactions and argue that using artificial agents as social stimuli can offer valuable insights into the role of mind attribution in social cognition.

## Impact Statement

Our findings provide novel evidence for a vicarious eye contact effect: eye contact elicits psychophysiological responses not only during dyadic interaction but also when observers witness gaze exchanges between others. Moreover, observers' psychophysiological responses differed when viewing eye contact between two humans versus between a human and a humanoid robot, suggesting that mind attribution could play a key role in third‐person social cognition.

## 1 Introduction

Eye contact is one of the key signals in human social interaction. It is diversely used to convey information, facilitate and regulate interactions, express emotions, and exercise social control (Foddy 1978; Jellison and Ickes 1974; Kendon 1967; Kimble and Olszewski 1980; for a review, see Kleinke 1986). As a central source of social information, eye contact elicits various cognitive, affective, and attentional responses in the observer (for reviews, see Conty et al. 2016; J. K. Hietanen 2018; Senju and Johnson 2009). Studies have associated eye contact with more favorable evaluations of another's likeability, attractiveness, and friendliness (Abele 1981; Argyle et al. 1974; Strick et al. 2008). Eye contact also quickly captures the observer's attention and sustains it over time (Conty et al. 2006; Senju et al. 2005; Senju and Hasegawa 2005; von Grünau and Anston 1995). Even neonates have been shown to look longer at faces displaying direct gaze (i.e., a gaze toward the infant) than at faces displaying averted gaze (Farroni et al. 2002, 2004). All these findings highlight the central role of eye contact in human interaction.

In many studies, the affect‐ and attention‐related responses to eye contact have been investigated using various psychophysiological measures (e.g., J. K. Hietanen et al. 2008; Nichols and Champness 1971; Pönkänen et al. 2011). Psychophysiological measures are generally regarded as providing more reliable insight into these processes than subjective measures for two reasons: First, compared to subjective measures, psychophysiological responses are less susceptible to response biases (e.g., the social desirability effect; see Kuncel and Tellegen 2009; Wood et al. 2016). Second, psychophysiological measures do not rely on participants' conscious responses and may, therefore, capture responses that are not consciously accessible. This is particularly useful for investigating responses to eye contact, the processing of which occurs partly below the conscious level (Rothkirch et al. 2015; Stein et al. 2011). In fact, self‐report measures of affect have yielded contradictory results, with some studies reporting more positive affective responses to eye contact than averted gaze (Chen et al. 2017; Uono and Hietanen 2015) and others reporting the opposite effect (J. K. Hietanen et al. 2008; Pönkänen et al. 2011). This inconsistency suggests that subjective affect‐related responses to eye contact are not robust and could easily be influenced by factors such as subtle differences in experimental design.

Psychophysiological studies have consistently shown that eye contact increases affective arousal, as indexed by greater skin conductance responses (SCRs), compared to averted gaze (Helminen et al. 2011; Jarick and Bencic 2019; Nichols and Champness 1971; Prinsen and Alaerts 2019). Skin conductance reflects the activity of the sympathetic nervous system and, being modulated by the amygdala (Laine et al. 2009; Mangina and Beuzeron‐Mangina 1996), is considered a robust indicator of affective arousal (Critchley 2002; Dawson et al. 2000; Fowles et al. 1981). Studies employing facial electromyography (fEMG) have shown that eye contact increases muscle activity in the zygomaticus area while decreasing activity in the corrugator area (Chen et al. 2024; J. K. Hietanen et al. 2018; J. K. Hietanen and Peltola 2021). This pattern of muscle activity change is considered to reflect positive affect (Cacioppo et al. 1986; Dimberg 1990; Larsen et al. 2003), suggesting that the affective response to eye contact is positively valenced. However, it is possible that facial expressions, especially during social interaction, reflect not only emotional reactions but also individuals' social motives (e.g., Buck 1994; Fridlund 1991; Parkinson 2005) and highly automatized affiliative responses (Hess et al. 2000; Knutson 1996; Niedenthal et al. 2010) to others' affiliative signals. Psychophysiological studies have further shown that eye contact, compared to averted gaze, elicits more intense momentary deceleration of heart rate (Akechi et al. 2013; Chen et al. 2024; J. O. Hietanen et al. 2020). A heart rate deceleration response (HRDR) is characterized by an initial decrease in heart rate followed by an increase toward the baseline and is considered to reflect attention orienting to external stimuli that are affectively and motivationally significant (Bradley 2009; Bradley et al. 1993; Graham and Clifton 1966).

Interestingly, previous studies have shown that eye contact with a humanoid robot elicits affect‐ and attention‐related psychophysiological responses similar to those evoked by eye contact with a human, including greater SCRs, increased zygomaticus activity, decreased corrugator activity, and more pronounced HRDRs when viewing the robot's direct versus averted gaze (Kiilavuori et al. 2021, 2022; Linnunsalo et al. 2023, 2024). Humanoid robots have been programmed to imitate human behavior—such as speech, facial expressions, and social gestures (Becker‐Asano and Ishiguro 2011; J. Li and Chignell 2011; Zecca et al. 2008)—leading humans to perceive them as entities possessing mind‐like characteristics, including intentionality, agency, and sentience (H. M. Gray et al. 2007; K. Gray and Wegner 2012; Thellman et al. 2017; Ward et al. 2013). Together, these findings suggest that despite being artificial, humanoid robots are attributed mind‐like characteristics to an extent that makes their direct gaze perceived as a socially relevant cue.

Almost all previous studies investigating psychophysiological responses to eye contact have focused on dyadic gaze interactions, that is, situations in which the observer is the target of another's attention. However, in real‐life multi‐person social interactions, individuals also observe eye contact between other people. Importantly, vicarious eye contact may evoke responses in the observer, even when they are not directly involved in the interaction. Studies have shown that individuals are more likely to follow the joint gaze direction of two people if those individuals first make eye contact with each other, compared to when they initially gaze away from each other (Böckler et al. 2011, 2014). Individuals also infer social intentions from observed eye contact. For example, when two people make eye contact, observers may interpret their speech as directed toward each other, which can diminish the observer's memory for the speech content (Lanthier et al. 2022). Given that observed eye contact between others serves as a meaningful social cue, it is essential to investigate whether it elicits affect‐ and attention‐related psychophysiological responses in the observer, akin to those observed during dyadic eye contact.

Research has shown that humans are able to adopt another individual's visual perspective, allowing them to consider how the other sees the environment (Furlanetto et al. 2013; Quesque et al. 2018; Todd et al. 2011; Tversky and Hard 2009). In fact, visual perspective taking is arguably a key component of human social interaction (Hamilton et al. 2009; Kampis and Southgate 2020; Wolbers and Hegarty 2010). Moreover, people may experience affective responses when observing others encountering emotionally salient situations (Niedenthal and Brauer 2012; Wondra and Ellsworth 2015). For example, people may experience embarrassment when they observe others in embarrassing situations (Hawk et al. 2011; Krach et al. 2011; Müller‐Pinzler et al. 2012) or anger when they observe others receiving unfair treatment (Hechler and Kessler 2018; Vitaglione and Barnett 2003). Vicarious affective responses offer valuable information to the observer, helping them adjust their behavior and facilitate social interaction (for reviews, see Galinsky et al. 2005; Skversky‐Blocq et al. 2021). For instance, vicariously experienced anger may motivate observers to help victims of unfair treatment (Vitaglione and Barnett 2003). The concept of vicarious emotions is closely related to affective empathy, that is, experiencing the emotions displayed by others (Singer and Lamm 2009; Walter 2012). However, some researchers have argued that vicarious emotions differ from affective empathy in that they can occur even when the observed person displays no emotions (see the review by Paulus et al. 2013). Vicarious emotions have been argued to stem from the interaction of two socio‐cognitive simulation processes: mirroring and mentalizing (Keysers and Gazzola 2007; Paulus et al. 2013; Waytz and Mitchell 2011). The former refers to the mapping of observed behaviors onto one's own nervous system (Rizzolatti and Sinigaglia 2010; Waytz and Mitchell 2011), whereas the latter refers to imagining oneself in the observed individual's position (Hein and Singer 2008; Keysers and Gazzola 2007).

To our knowledge, only one previous study has attempted to investigate psychophysiological responses to vicarious eye contact. In a study by Sun et al. (2023), participants viewed videos of two people either making eye contact with each other or displaying unreciprocated gaze (i.e., one of the two people gazing toward the other but the other gazing downward). While participants viewed these videos, their affect‐ and attention‐related psychophysiological responses (SCR for affective arousal, fEMG for affective valence or affiliative responses, and HRDR for attention orienting) were measured. To increase the impression of genuine social interaction, participants were led to believe that they were participating in a real‐time video call with the two gaze models. However, the results did not provide evidence for a vicarious eye contact effect, as none of the psychophysiological responses differed between the vicarious eye contact and non‐eye contact conditions. One possible explanation for this null finding is that, despite participants being led to believe they were engaged in real‐time interaction with the two gaze models, the lack of a shared physical environment may have rendered the vicarious eye contact socially irrelevant.

In the present preregistered study (see https://osf.io/q7vhs/overview?view_only=ad06aab2b7b14a009e75226c32961dbe), we investigated whether vicarious eye contact elicits affect‐ and attention‐related psychophysiological responses in the observer. Importantly, in contrast to Sun et al. (2023), we investigated these responses during live interaction, in which the participant and the two stimulus people shared the same physical environment. In the first experiment, participants viewed vicarious eye contact and non‐eye contact conditions between two people while their SCRs, fEMG responses, and HRDRs were measured. In the second experiment, we investigated vicarious responses to a less inherently social gaze interaction: eye contact between a natural agent (a human) and an artificial agent (a humanoid robot). Building on the first experiment, which focused on eye contact between two humans, the second study examined a new form of social interaction that is rapidly becoming more common in everyday life. The second experiment was otherwise similar to the first, but participants viewed eye contact and non‐eye contact conditions between a human and a humanoid robot while their psychophysiological responses were measured.

## 2 Experiment 1

To investigate affect‐ and attention‐related psychophysiological responses to vicarious eye contact, we showed participants various gaze interactions between two human confederates seated face‐to‐face directly in front of the participants. Participants were shown four types of gaze interactions: both confederates gazing into each other's eyes (eye contact), both confederates gazing away from each other (averted gaze), and two unreciprocated gaze conditions in which one confederate gazed at the other's eyes while the other looked away. Furthermore, we designated one of the two confederates as the person‐of‐interest (POI) and instructed participants to focus particularly on that individual. This setup enabled us to investigate potential asymmetries between the two latter conditions: one in which the POI gazed at the other confederate, who looked away (attempted eye contact), and another in which the POI looked away while the other confederate gazed at the POI (avoided eye contact). To maintain precise control over stimulus presentation in this live procedure, a voltage‐sensitive liquid crystal window (see Section 2.1.2) was placed between the participant and the confederates. The window was switched between transparent and opaque to control stimulus visibility. Importantly, to compare psychophysiological responses to vicarious and dyadic eye contact, we also measured participants' responses to a single confederate's direct gaze (toward the participant) and averted gaze (away from the participant) in a separate experimental block.

In both the vicarious and dyadic standpoints, we expected greater SCRs, increased zygomaticus activity, decreased corrugator activity, and more pronounced HRDRs in response to eye contact relative to non‐eye contact conditions. Non‐eye contact conditions included averted gaze, attempted eye contact, and avoided eye contact in the vicarious standpoint, and averted gaze in the dyadic standpoint. We also expected that these responses would be greater for dyadic than vicarious eye contact.

### 2.1 Methods

#### 2.1.1 Participants

The participants were 110 adults (19–75 years old, *M*_age_ = 35.45 years, SD_age_ = 14.16; *N*_women_ = 76, *N*_men_ = 33, *N*_other_ = 1) with no reported history of neurological or psychiatric disorders. All participants had normal or corrected‐to‐normal vision. A power analysis conducted with G*Power (Faul et al. 2007) indicated that our sample size was sufficiently large to reliably detect gaze effects of *d*_z_ ≥ 0.27 (1 − *β* = 0.80, *α* = 0.05). We excluded some participants from the statistical analyses because of poor‐quality data, resulting in final sample sizes of 103 for SCR analyses, 109 for zygomaticus fEMG analyses, 106 for corrugator fEMG analyses, and 101 for HRDR analyses.

All data were collected at the Human Information Processing Laboratory (Tampere, Finland) between June and November 2023. Participants were recruited using the news feed and email lists of Tampere University, an undergraduate course in psychology, and the Tampere University DMlab pool (Greiner 2015). All participants received a movie ticket, a restaurant gift card, or partial course credit as a token of participation. All participants provided written, informed consent before the study was initiated. The Ethics Committee of the Tampere region reviewed the study protocol and gave a favorable statement about it.

#### 2.1.2 Stimuli and Procedure

##### 2.1.2.1 Vicarious Block

**FIGURE 1:**
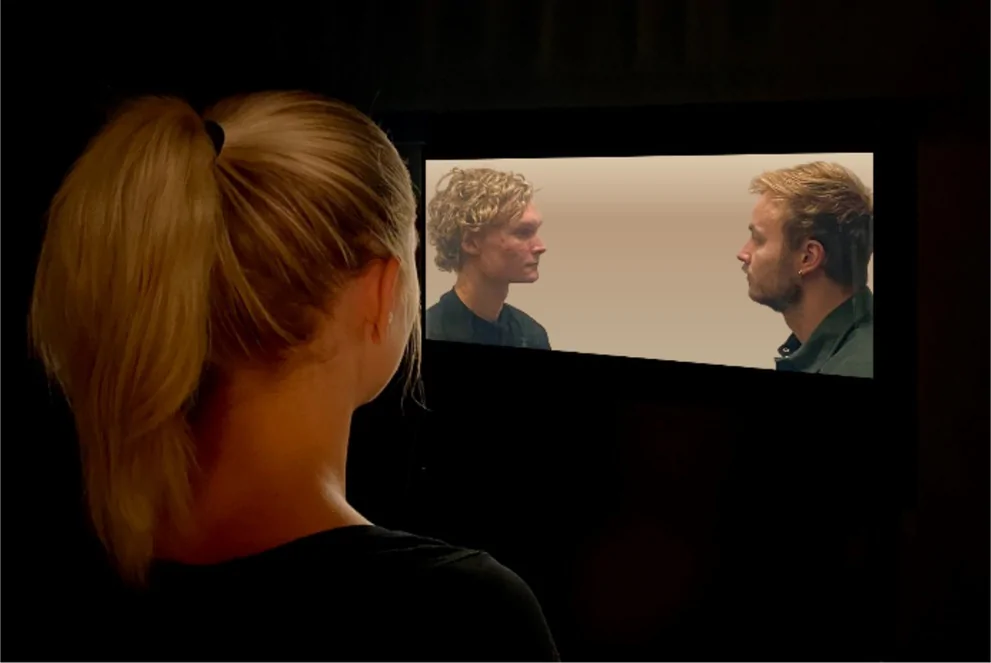
A participant viewing two confederates through a voltage‐sensitive liquid crystal window. Note that in the experiment, the confederates were of the same gender as the participant.

**FIGURE 2:**
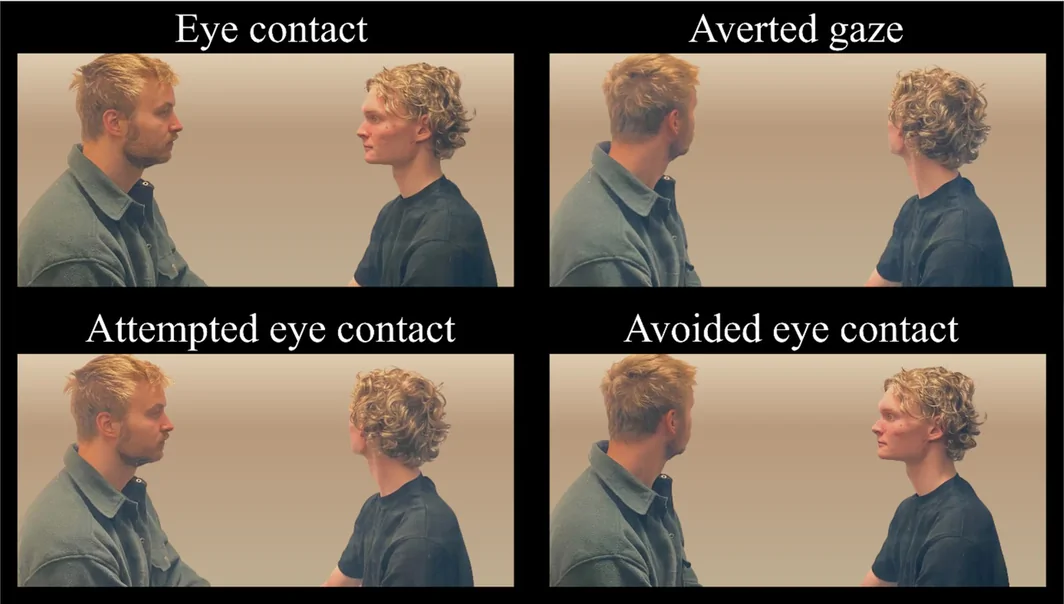
Illustration of the four stimulus conditions from a third‐person perspective. In this illustration, the person on the left represents the person‐of‐interest (POI).

The stimuli featured two research assistants of the same gender as the participant. These confederates were carefully trained to maintain a still, neutral facial expression while maintaining a slight muscle tonus in the lower face area to avoid looking fatigued. The confederates were allowed to blink naturally. They adopted gaze conditions by rotating their whole head to make their gaze direction evident to the participant (see Figures 1 and 2). The confederates were presented to the participant through a voltage‐sensitive liquid crystal (LC) window (NSG UMU Products Co. Ltd.) embedded in a black wall. The LC window could be switched between transparent (allowing the participant to see the two confederates) and opaque (blocking the view) states with a negligible delay of 3 ms, enabling precise temporal control of stimulus presentation. The experimenter controlled the state of the LC window (transparent or opaque) using E‐Prime software (v2.0, Psychology Software Tools Inc.) running on a desktop computer. Both the participants and the two confederates were seated approximately 60 cm from the LC window. Figure 1 illustrates the setup.

At the beginning of the study, the experimenter and the two confederates introduced themselves to the participant. The experimenter then attached the measurement electrodes and seated the participant at the LC window. The confederates sat on the other side of the LC window facing each other. The LC window measured 23 × 57.5 cm, which was large enough to display both confederates. The experimenter designated one of the confederates as the person‐of‐interest (POI) and instructed the participant to consider how the POI might be feeling during the stimulus trials. The location of the POI, on either the left or right side of the participant's field of view, was counterbalanced across participants. After the participant confirmed understanding of the instructions, the experimenter turned off the lights in the participant's area, moved to the monitoring area (separated from the participant's area by a curtain), and initiated the stimulus trials.

The experimenter monitored the participant's skin conductance level and, once it had stabilized for a few seconds, manually initiated a stimulus trial by switching the LC window to a transparent state for 3000 ms. The transparent LC window revealed one of four possible gaze conditions. (1) In *eye contact*, the POI looked directly into the eyes of the other confederate who reciprocated by looking into the POI's eyes. (2) In *averted gaze*, neither the POI nor the other confederate looked at each other; instead, both directed their gaze toward the back wall. (3) In *attempted eye contact*, the POI looked at the other confederate's eyes, while the other confederate looked at the back wall. (4) In *avoided eye contact*, the POI looked at the back wall, whereas the other confederate directed their gaze toward the POI's eyes. These four gaze conditions are illustrated in Figure 2. Each gaze condition was shown six times (24 trials in total in the vicarious block), and the order of presentation was counterbalanced such that no more than two consecutive presentations of the same gaze condition occurred. The intervals between stimulus trials were at least 15 s. During these inter‐stimulus intervals, invisible to the participant, the confederates were informed about the next gaze condition on a computer screen and adopted the required gaze direction before the experimenter initiated the next trial.

##### 2.1.2.2 Dyadic Block

After the 24 trials in the vicarious block, the experimenter readjusted the visible area of the LC window to 23 × 26 cm and informed the participant that the POI had left the room and that only one confederate remained on the other side of the LC window. The experimenter instructed the participant to simply look at the confederate during the subsequent trials.

**FIGURE 3:**
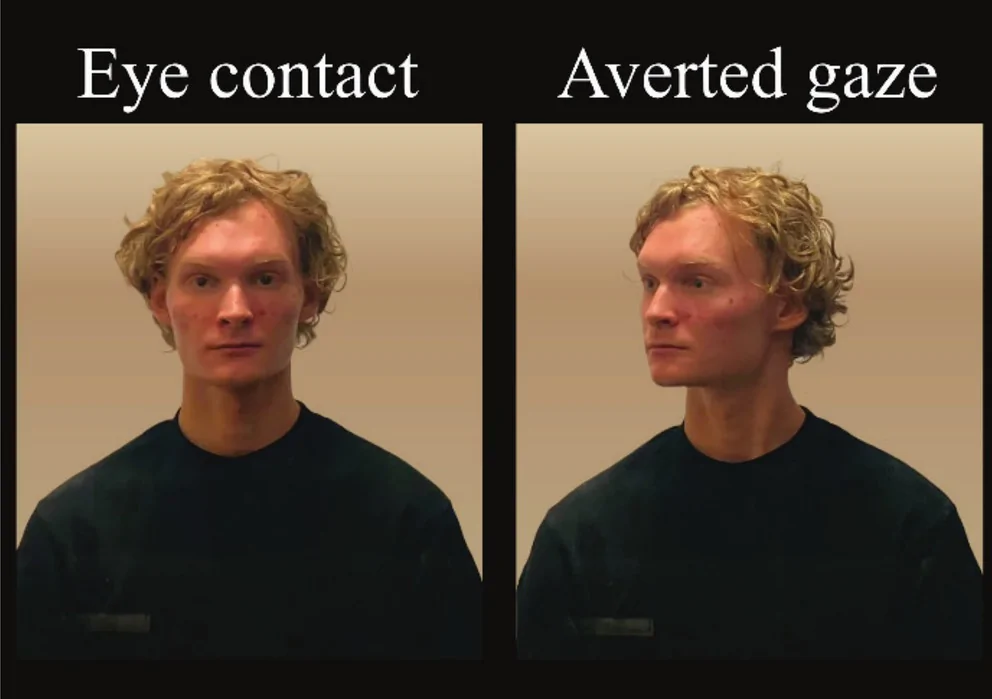
Illustration of the two gaze conditions in the dyadic block. Averted gaze stimuli were counterbalanced across the left and right gaze directions.

The stimulus trials in the dyadic block were conducted similarly to those in the vicarious block. However, there were only two possible gaze conditions: (1) In *eye contact*, the confederate looked directly into the participant's eyes. (2) In *averted gaze*, the confederate looked (and rotated their head) 65 degrees to the left or right (counterbalanced). These two gaze conditions are illustrated in Figure 3. The dyadic block consisted of 12 stimulus trials (6 for eye contact, 6 for left/right averted gaze), semi‐randomized to ensure that no more than four consecutive trials of the same gaze condition could occur. As in the vicarious block, each stimulus trial lasted 3000 ms, and inter‐trial intervals were at least 15 s.

After the 12 trials in the dyadic block, the experimenter removed the electrodes from the participant and asked them to provide a written response to the following open‐ended question: “Think about the block where two people were behind the window. What kinds of interactions or situations did you notice between them?”. This question served as a manipulation check for the vicarious block, assessing whether participants had perceived instances of eye contact and non‐eye contact between the two confederates. We did not include a corresponding manipulation check for the dyadic block as the dyadic gaze direction manipulation has been validated in several previous studies (for an identical gaze manipulation paradigm, see Kiilavuori et al. 2021, 2022). After completing the manipulation check, the participant was debriefed and rewarded for their participation.

#### 2.1.3 Data Acquisition and Analysis

All physiological signals were continuously recorded at a sampling rate of 1000 Hz using BrainVision Recorder software (v1.24, Brain Products GmbH). The signals were amplified with an actiCHamp Plus amplifier (Brain Products GmbH). An Ag/AgCl ground electrode was attached to the participant's forehead just below the hairline. All physiological signals were measured and post‐processed following established practices in the field.

##### 2.1.3.1 Skin Conductance

Skin conductance was measured with two Ag/AgCl electrodes containing isotonic paste, attached to the index and middle fingers of the participant's left hand. The signal was post‐processed using BrainVision Analyzer software (v2.3, Brain Products GmbH). The signal was re‐sampled to 100 Hz and high‐cutoff filtered at 10 Hz. An SCR was defined as the maximum change in skin conductance within a 900‐ to 6000‐ms post‐stimulus period, which was calculated by first identifying the lowest skin conductance within a 900‐ to 3500‐ms post‐stimulus period and then subtracting it from the subsequent highest skin conductance within a 900‐ to 6000‐ms post‐stimulus period. Even if a trial contained multiple SCRs, only the first was analyzed. Any SCRs that were smaller than 0.01 μS were re‐coded as 0 μS but were nevertheless included in the analyses. Trials that exhibited a skin conductance increase of at least 0.01 μS during the 0‐ to 900‐ms post‐stimulus period were rejected from the analysis (12.02% of trials). Participants with more than half of the trials (4 or more) rejected in any stimulus condition (regardless of standpoint) were excluded from the analyses (7 participants). Finally, a log10(SCR + 1) transformation was conducted on the SCRs.

##### 2.1.3.2 Facial Electromyography

Facial electromyography was measured with two pairs of gelled Ag/AgCl electrodes that were attached 1 cm apart to the participant's left cheek (zygomaticus) and just above the participant's left eyebrow (corrugator). The electrodes were placed according to the guidelines by Fridlund and Cacioppo (1986). The zygomaticus and corrugator signals were post‐processed using BrainVision Analyzer. Both signals were band‐pass filtered at 28–249 Hz and notch filtered at 50 Hz, after which the signals were rectified. Unexpectedly, even after applying these filters, the signals remained notably noisy around stimulus onset and offset. To reduce the influence of these noise peaks, we applied the Interpolate Time Range procedure to linearly interpolate the signal within two specific time windows. For the zygomaticus data, the time windows were −350 to 350 ms and 2650 to 3350 ms. For the corrugator data, the interpolated time windows were −100 to 100 ms and 2900 to 3000 ms. These time ranges were determined based on visual inspections of the data.

Next, we had planned to assign a time window of −500 to 0 ms as a baseline period. However, for the zygomaticus data, the time window of interpolation covered most of the planned baseline period. Therefore, we selected the interval from −850 to −350 ms as the baseline period for the zygomaticus data, instead of the −500 to 0 ms time window that was used for the corrugator data. For each trial, the baseline period was visually inspected, and trials with a notable change in muscle activity during the baseline were rejected from the analysis as artifacts (2.85% of trials for zygomaticus and 6.64% of trials for corrugator). If more than half of the trials were rejected in any stimulus condition, the participant was excluded from the analysis concerning that muscle area (1 participant for zygomaticus and 4 participants for corrugator). The trials were segmented into 500‐ms epochs, beginning with the baseline period (−850 to −350 ms before stimulus onset for zygomaticus and −500 to 0 ms for corrugator), followed by six consecutive epochs spanning 0 to 3000 ms after stimulus onset. The signal values were averaged within each 500‐ms epoch, resulting in a baseline value and six post‐stimulus values (epochs 1–6). For each participant, these values were standardized across stimulus conditions, separately for zygomaticus and corrugator signals. Finally, fEMG change scores were calculated per trial by subtracting the standardized baseline value from each standardized post‐stimulus epoch.

##### 2.1.3.3 Heart Rate Deceleration

We measured electrocardiography with two gelled Ag/AgCl electrodes that were attached below the participant's collarbones. Offline, we first used a Matlab‐based algorithm to detect R peaks in the recorded signal and then manually corrected any missing or falsely identified R peaks. Any trials with excessive noise (preventing reliable identification of R peaks) were rejected from the analysis (8.61% of trials). Participants with more than half of the trials rejected in any stimulus condition were excluded from the analysis (9 participants). The intervals between R peaks (inter‐beat intervals, IBIs) were quantified and averaged within 500‐ms epochs from 500 ms before the stimulus onset (baseline) to 6000 ms after the stimulus onset (epochs 1–12). This 6000‐ms post‐stimulus time window is commonly used to capture the typical duration of an HRDR. The IBIs were converted to beats‐per‐minute (BPM = 60,000/IBI). HRDRs were calculated by subtracting the baseline BPM from each post‐stimulus epoch BPM.

##### 2.1.3.4 Statistical Analyses

Statistical analyses were conducted with trial‐by‐trial linear mixed‐effects models (LMMs) using R (v4.2.2; R Core Team 2022) and lme4 (v1.1.34; Bates et al. 2015). Psychophysiological responses in the vicarious block were analyzed by including gaze (eye contact vs. averted gaze vs. attempted eye contact vs. avoided eye contact) as a fixed effect. Additionally, for fEMG and HRDR analyses, time (1–6 for fEMG and 1–12 for HRDR) and the Gaze × Time interaction were included as fixed effects. Regarding the random effects structure, we began by including a by‐participant random intercept and random slopes for all fixed effects and their interactions (Barr et al. 2013). If the model failed to converge due to insufficient data support, we gradually simplified the structure by first removing the random slope for the interaction term, then the random slope for time, and finally the random slope for gaze, until the model converged successfully. Additionally, we removed any random slopes that resulted in a singular fit. The analysis code is available at https://osf.io/mpds7/overview?view_only=2490e4e0bac34fa0944be7b3a52e030b. For the dyadic block, LMMs were created similarly, but the fixed effect of gaze had only two levels (eye contact vs. averted gaze) instead of four. The fixed effects were assessed using Type III analyses of variance (ANOVAs) via the *lmerTest* package (v3.1.3; Kuznetsova et al. 2017), with degrees of freedom estimated using Satterthwaite's approximation. For statistically significant effects, pairwise comparisons were conducted using the *emmeans* package (v1.8.8; Lenth 2023). These pairwise comparisons were not corrected for multiple testing.

If we observed similar gaze effects (i.e., contrasts between the eye contact and non‐eye contact conditions) in the vicarious and dyadic blocks, we compared the effects by creating an LMM with gaze (eye contact vs. averted gaze), standpoint (vicarious vs. dyadic), and the Gaze × Standpoint interaction as fixed effects and the maximal random‐effects structure described above. To avoid creating unnecessarily complex models, the time factor (fEMG and HRDR) was omitted by averaging the responses over time epochs within a trial. For the vicarious standpoint, responses to averted gaze, attempted eye contact, and avoided eye contact were averaged to reduce the number of levels to two (eye contact and averted gaze) enabling comparison between the vicarious and dyadic standpoints.

#### 2.1.4 Deviations From the Preregistered Analysis Plan

We preregistered the use of a baseline window from −500 to 0 ms before stimulus onset for the fEMG and HRDR analyses. However, as described above, we used a baseline window from −850 to −350 ms for the zygomaticus analyses due to the interpolation procedure. We also had not preregistered the use of linear interpolation in the fEMG preprocessing, as the notable noise peaks were not anticipated. Finally, our preregistration specified LLMs with random intercepts for participant number and trial number (1–6), without random slopes. However, we later decided to include by‐participant random intercepts and random slopes for all fixed effects and their interactions, as this approach is recommended to reduce the risk of Type I errors (Barr et al. 2013).

### 2.2 Results

#### 2.2.1 Manipulation Check

All participants reported observing instances of eye contact and instances of no eye contact between the two confederates in the vicarious block. Interestingly, 52.7% of participants also attributed affective connotations to the observed gaze conditions. Participants attributed, for example, pleasant senses of connection, presence, and empathy to vicarious eye contact and unpleasant senses of disregard, disappointment, and anger to non‐eye contact conditions (averted gaze, attempted eye contact, and avoided eye contact).

#### 2.2.2 Skin Conductance Results

##### 2.2.2.1 Vicarious Standpoint

**FIGURE 4:**
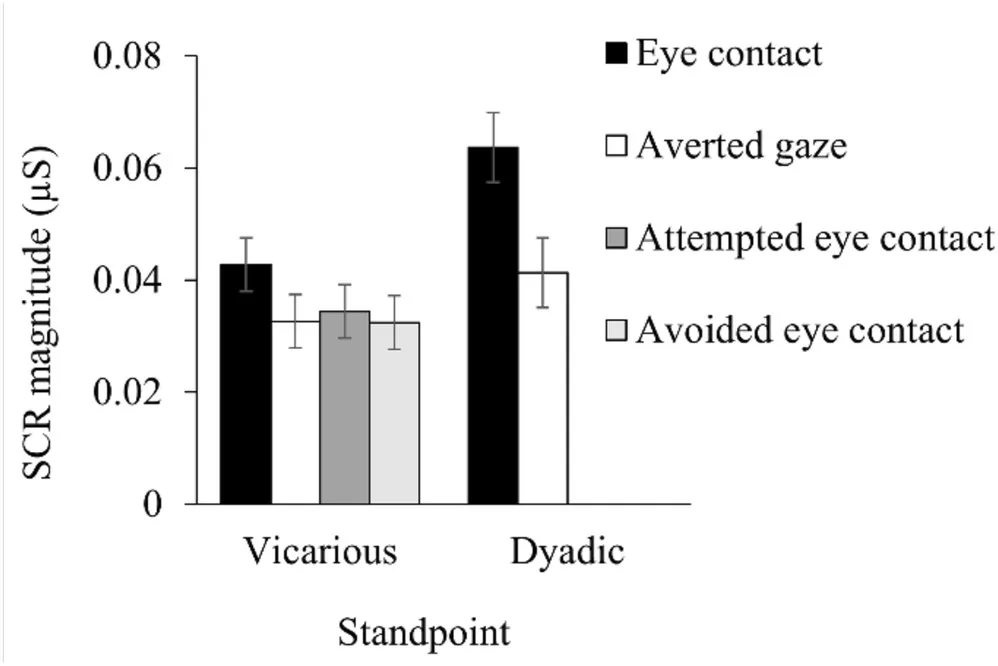
Mean skin conductance responses across gaze conditions, shown separately for the vicarious standpoint (participant viewing two persons) and the dyadic standpoint (participant viewing one person). Error bars represent the standard error. Response values were log10‐transformed.

The results showed a statistically significant main effect of gaze (*F*_(3, 2095.1)_ = 3.81, *p* = 0.010), indexing greater SCRs to eye contact (*M* = 0.043 μS, SEM = 0.0048) than averted gaze (*M* = 0.033 μS, SEM = 0.0048, *t*_(2095)_ = 2.85, *p* = 0.004, *d*_z_ = 0.281), attempted eye contact (*M* = 0.034 μS, SEM = 0.0048, *t*_(2095)_ = 2.36, *p* = 0.018, *d*_z_ = 0.233), and avoided eye contact (*M* = 0.032 μS, SEM = 0.0048, *t*_(2095)_ = 2.93, *p* = 0.003, *d*_z_ = 0.289). The SCR results are illustrated in Figure 4.

##### 2.2.2.2 Dyadic Standpoint

The results showed a main effect of gaze (*F*_(1, 986.9)_ = 22.75, *p* < 0.001), indexing greater SCRs to eye contact (*M* = 0.064 μS, SEM = 0.0063) than averted gaze (*M* = 0.041 μS, SEM = 0.0062, *d*_z_ = 0.470).

##### 2.2.2.3 Comparison Between the Vicarious and Dyadic Standpoints

A statistically significant main effect of gaze (*F*_(1, 105.1)_ = 20.79, *p* < 0.001) indicated more intense SCRs to eye contact (*M* = 0.054 μS, SEM = 0.0062) than averted gaze (*M* = 0.037 μS, SEM = 0.0043, *d*_z_ = 0.449). There was also a main effect of standpoint (*F*_(1, 106.4)_ = 18.58, *p* < 0.001), indicating more intense SCRs in the dyadic (*M* = 0.053 μS, SEM = 0.0058) versus vicarious standpoint (*M* = 0.038 μS, SEM = 0.0046, *d*_z_ = 0.425). Importantly, there was a statistically significant Gaze × Standpoint interaction (*F*_(1, 252.6)_ = 6.83, *p* = 0.009). Pairwise comparisons showed that SCRs were greater to dyadic eye contact (*M* = 0.065 μS, SEM = 0.0075) compared to vicarious eye contact (*M* = 0.043 μS, SEM = 0.0057, *t*_(109)_ = 4.55, *p* < 0.001, *d*_z_ = 0.449). SCRs did not differ between dyadic and vicarious averted gaze (*p* = 0.094).

#### 2.2.3 Facial Electromyography Results

##### 2.2.3.1 Vicarious Standpoint

**FIGURE 5:**
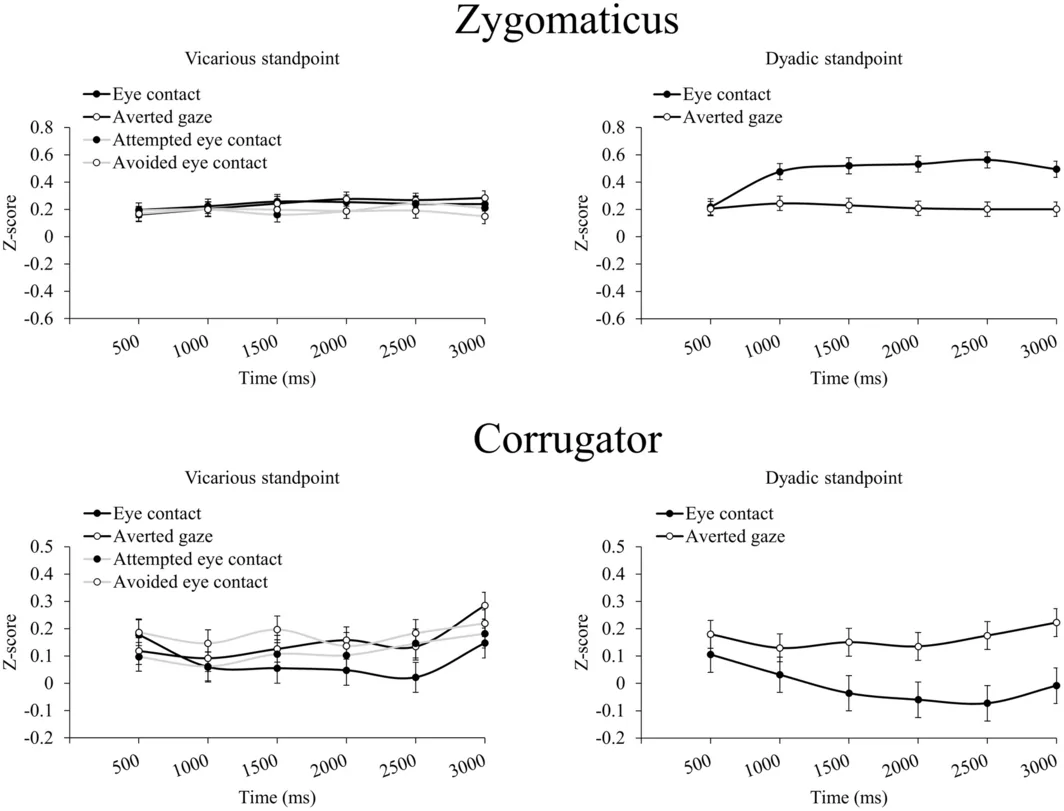
Mean zygomaticus and corrugator activity changes across gaze conditions, presented separately for the vicarious standpoint (participant viewing two persons) and the dyadic standpoint (participant viewing one person). Error bars represent the standard error. Response values were standardized.

For zygomaticus, the main effects of gaze and time and their interaction were all statistically nonsignificant (all *p*s ≥ 263). For corrugator, there was a main effect of time suggesting an increase of muscle activity as a function of time (*F*_(5, 14,370.4)_ = 4.74, *p* < 0.001). The main effect of gaze and the Gaze × Time interaction were statistically nonsignificant (both *p*s ≥ 0.194). The fEMG results are illustrated in Figure 5.

##### 2.2.3.2 Dyadic Standpoint

For zygomaticus, a statistically significant main effect of gaze (*F*_(1, 105.5)_ = 30.54, *p* < 0.001) suggested that muscle activity was greater in response to eye contact (*M* = 0.47, SEM = 0.045) than averted gaze (*M* = 0.22, SEM = 0.037, *d*_z_ = 0.529). There was also *a* main effect of time (*F*_(5, 7377.0)_ = 4.76, *p* < 0.001), indicating a change in muscle activity as a function of time. More specifically, a statistically significant Gaze × Time interaction (*F*_(5, 7377.0)_ = 4.47, *p* < 0.001) suggested that muscle activity increased over time in response to eye contact but not in response to averted gaze. For corrugator, the results showed a main effect of gaze (*F*_(1, 105.3)_ = 8.33, *p* = 0.005), indicating that muscle activity was greater in response to averted gaze (*M* = 0.17, SEM = 0.039) than eye contact (*M* = −0.0067, SEM = 0.056, *d*_z_ = 0.280). There was also a main effect of time (*F*_(5, 7146.6)_ = 2.33, *p* = 0.040), indicating a decrease in overall muscle activity as a function of time. The Gaze × Time interaction was not statistically significant (*p* = 0.096).

#### 2.2.4 Heart Rate Deceleration Results

##### 2.2.4.1 Vicarious Standpoint

**FIGURE 6:**
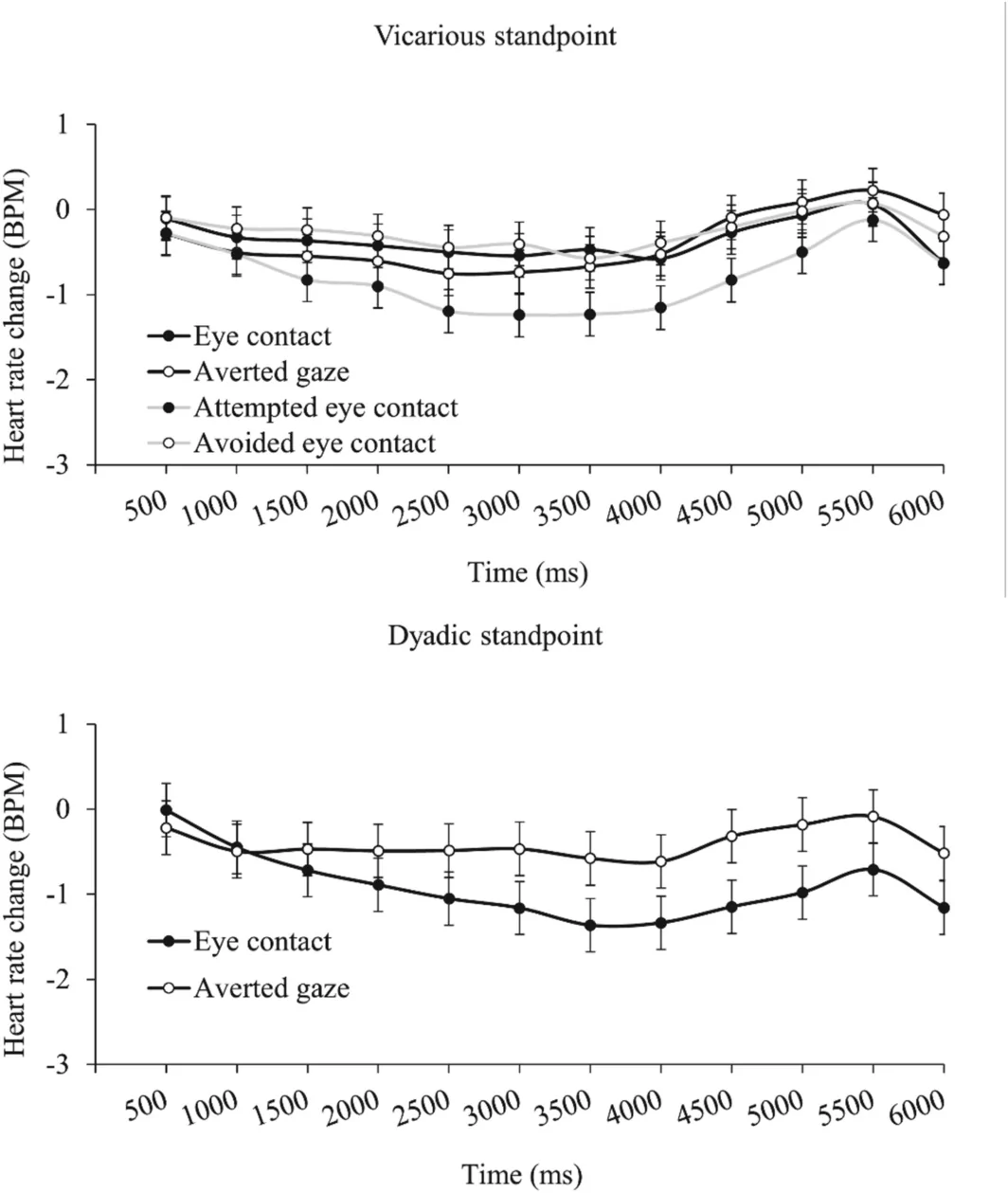
Mean heart rate changes across gaze conditions, shown separately for the vicarious standpoint (participant viewing two persons) and the dyadic standpoint (participant viewing one person). Error bars represent the standard error.

The main effect of gaze was statistically significant (*F*_(3, 28,773)_ = 11.74, *p* < 0.001). Interestingly, heart rate deceleration was more intense in response to attempted eye contact (*M* = −0.79 BPM, SEM = 0.12) than eye contact (*M* = −0.35 BPM, SEM = 0.12, *t*_(28,773)_ = 4.52, *p* < 0.001, *d*_z_ = 0.449), averted gaze (*M* = −0.37 BPM, SEM = 0.12, *t*_(28,774)_ = 4.28, *p* < 0.001, *d*_z_ = 0.426), or avoided eye contact (*M* = −0.26 BPM, SEM = 0.12, *t*_(28,772)_ = 5.43, *p* < 0.001, *d*_z_ = 0.540). The responses to eye contact did not differ from the responses to averted gaze or avoided eye contact (both *p*s ≥ 0.363). A statistically significant main effect of time (*F*_(11, 28,772)_ = 4.82, *p* < 0.001) indexed a heart rate deceleration. The Gaze × Time interaction was statistically nonsignificant (*p* = 0.999). The HRDR results are illustrated in Figure 6.

##### 2.2.4.2 Dyadic Standpoint

Surprisingly, the main effect of gaze and the Gaze × Time interaction were statistically nonsignificant (both *p*s ≥ 0.137). There was only a main effect of time (*F*_(11, 14,284)_ = 2.31, *p* = 0.008) reflecting heart rate deceleration.

### 2.3 Discussion

As hypothesized, observing eye contact between two people elicited a vicarious eye contact effect. Participants' SCRs were greater during vicarious eye contact than during non‐eye contact conditions (averted gaze, attempted eye contact, and avoided eye contact). However, the fEMG results did not differ between these conditions. Prior research has shown that interactions between individuals elicit stronger fEMG patterns indicative of positive affect (increased zygomaticus activity and decreased corrugator activity) than non‐interactions, and that these fEMG patterns are stronger during positive than negative interactions (Balconi and Canavesio 2013; Balconi and Pagani 2015; Mota et al. 2021). It is possible that, in the present study, participants may not have interpreted the confederates' eye contact as clearly positive or affiliative, which could explain the absence of fEMG effects. Unlike typical social interactions, the confederates did not speak or display affiliative expressions, leaving the interaction—including the eye contact condition—as ambiguous or potentially tense to some participants. This lack of clear social or emotional context may have reduced the expected modulation of zygomaticus and corrugator activity.

Contrary to our hypothesis, HRDRs were not greater during vicarious eye contact than during non‐eye contact conditions, suggesting that vicarious eye contact does not capture attention in the same way as dyadic eye contact. Instead, attempted eye contact elicited the greatest HRDRs across all conditions, indicating that participants' attention was most strongly captured when the POI tried to initiate eye contact while the other confederate looked away. One explanation is that participants interpreted the non‐POI confederate's averted gaze as signaling social exclusion (see Leng et al. 2018; Wirth et al. 2010; Yang et al. 2023). Because humans are highly sensitive to cues of exclusion (Williams 2009), such situations may have triggered stronger attentional orienting.

For the dyadic standpoint, we replicated earlier findings showing that direct gaze (i.e., eye contact) elicits greater SCRs, increased zygomaticus activity, and decreased corrugator activity compared to viewing another person's averted gaze. Surprisingly, and in contrast to many previous studies (Akechi et al. 2013; Kiilavuori et al. 2021; Myllyneva and Hietanen 2015), we found no statistically significant difference in HRDRs between direct and averted gaze. This absence of an effect could be related to features of our experimental design: participants viewed the stimulus person's direct and averted gaze only *after* having already seen the same person during the vicarious gaze trials. Because HRDR amplitude typically declines with repeated exposure, and substantial changes in stimulus characteristics are usually required for recovery (see, e.g., Zimmer and Richter 2023), these repeated presentations may have reduced cardiovascular responsiveness. Supporting this interpretation, the HRDRs to dyadic eye contact in the present study were markedly smaller than those reported in earlier work (e.g., Akechi et al. 2013; Myllyneva and Hietanen 2015). Thus, habituation may have progressed to the point at which HRDRs no longer differentiated between direct and averted gaze.

## 3 Experiment 2

In the second experiment, we investigated whether the vicarious eye contact effect also occurs when observing eye contact between a human and a humanoid robot. Using the same setup as in Experiment 1, we presented participants with vicarious eye contact and non‐eye contact conditions between a human and a humanoid robot NAO. The human was always assigned as the POI. As in the first experiment, we conducted two experimental blocks: a vicarious eye contact block and a dyadic eye contact block.

We had preregistered the same hypotheses as in Experiment 1. In both the vicarious and dyadic standpoints, we expected greater SCRs, increased zygomaticus activity, decreased corrugator activity, and more pronounced HRDRs during eye contact than during non‐eye contact conditions (averted gaze, attempted eye contact, and avoided eye contact). We also expected direct (dyadic) eye contact with NAO to elicit stronger responses than observing (vicarious) eye contact between a human and NAO.

### 3.1 Methods

#### 3.1.1 Participants

We collected a new sample of 113 adult participants (18–75 years old, *M*_age_ = 37.91 years, SD_age_ = 15.71; *N*_women_ = 78, *N*_men_ = 35) with no reported history of neurological or psychiatric disorders and normal or corrected‐to‐normal vision. Our new sample did not differ statistically significantly from the sample in Experiment 1 by age or gender distribution (both *p*s ≥ 0.220). The new sample was also similar to the sample in Experiment 1 in terms of statistical power. After excluding participants because of poor‐quality data, we had a final sample size of 101 for SCR analyses, 112 for zygomaticus fEMG analyses, 112 for corrugator fEMG analyses, and 107 for HRDR analyses. Participants were recruited as described in Experiment 1, including obtaining a favorable statement from The Ethics Committee of the Tampere region.

#### 3.1.2 Stimuli and Procedure

The stimuli were a human research assistant of the same gender as the participant (as described in Experiment 1) and the humanoid robot NAO (Aldebaran SAS). NAO is a programmable, embodied robot capable of humanlike movements and speech. The behavior of NAO was programmed using Choregraphe software and the Python API for Aldebaran robots (Aldebaran SAS). Whereas the human confederate was allowed to blink naturally, NAO was programmed to blink every 3000 ms by briefly switching off its eye LEDs. As in Experiment 1, the stimuli were shown through the LC window to ensure precise control over the stimulus presentation duration.

##### 3.1.2.1 Vicarious Block

Again, the study consisted of a vicarious block and a subsequent dyadic block. At the beginning of the study, the experimenter introduced themselves to the participant, after which NAO and the human confederate were introduced. NAO was revealed in a sitting position, from which it autonomously stood up and greeted the participant. NAO said (in Finnish): “Hi, I'm NAO!” and performed some humanlike gestures such as head nods. The experimenter asked the participant to move around to demonstrate that NAO could follow the participant with its gaze (by rotating its head).

After this introduction, the experimenter attached the measurement electrodes and seated the participant at the LC window. The human confederate and NAO sat on the other side of the LC window facing each other. The experimenter asked the participant to consider how the human confederate might be feeling during the stimulus trials. The location of the human confederate (left or right) was counterbalanced across participants. After the participant confirmed understanding of the instructions, the experimenter turned off the lights in the participant's area and initiated the stimulus trials from the monitoring room.

**FIGURE 7:**
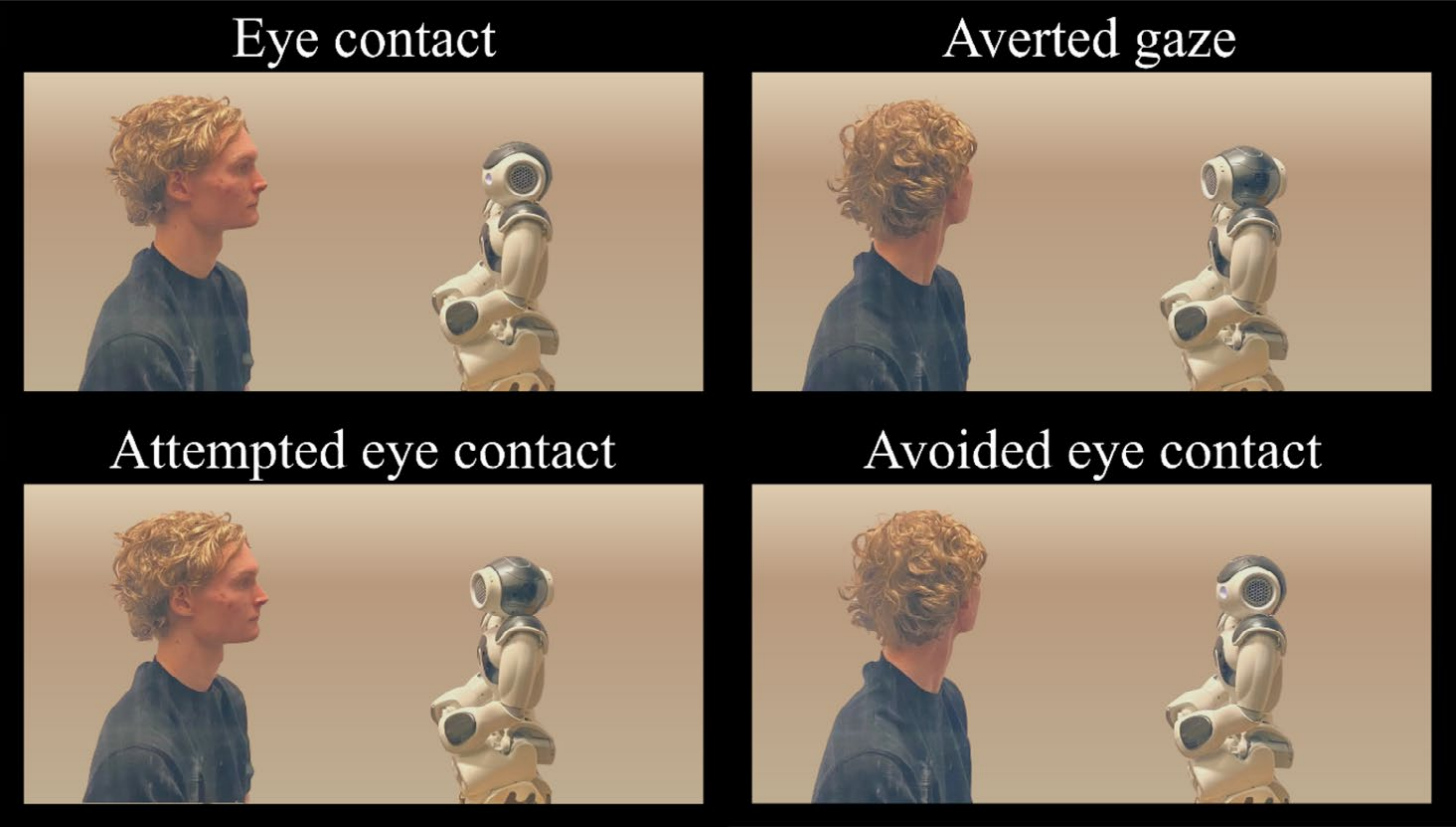
The four gaze conditions in the vicarious block.

The stimulus trials were conducted as described in Experiment 1. Again, the block consisted of four gaze conditions presented in a counterbalanced order: (1) In *eye contact*, the human confederate looked directly into NAO's eyes and NAO looked into the human confederate's eyes. (2) In *averted gaze*, the human confederate and NAO looked at the back wall. (3) In *attempted eye contact*, the human confederate looked at NAO's eyes, while NAO looked at the back wall. (4) In *avoided eye contact*, the human confederate looked at the back wall, while NAO looked at the human confederate's eyes. These gaze conditions are illustrated in Figure 7. Between trials, the experimenter and the human confederate were informed about the next stimulus condition on a computer screen, and the experimenter rotated NAO's head accordingly using the Python API running on a laptop.

##### 3.1.2.2 Dyadic Block

**FIGURE 8:**
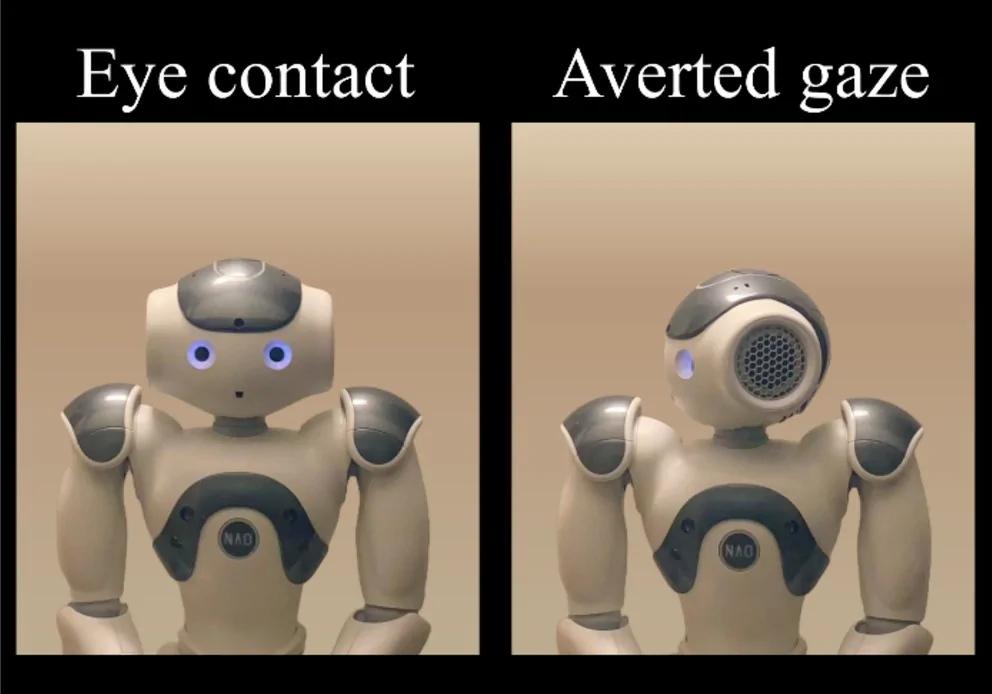
The eye contact and averted gaze conditions in the dyadic block.

After the vicarious block, the experimenter readjusted the visible area of the LC window to 23 × 26 cm and informed the participant that the human confederate had left the room and that now only NAO remained on the other side of the LC window. The experimenter instructed the participant to simply gaze at NAO during the subsequent trials. The stimulus trials (consisting of direct and averted gaze conditions) were then conducted as described in Experiment 1. The direct and averted gaze conditions of NAO are illustrated in Figure 8. After these stimulus trials, the experimenter removed the measurement electrodes from the participant and asked them to provide a written response to the following manipulation check question: “Think about the block in which two individuals were behind the window. What kinds of interactions or situations did you notice between them?” Again, this manipulation check concerned the vicarious block, and a corresponding manipulation check was not included for the dyadic block, as several previous studies have validated the dyadic eye contact manipulation paradigm using NAO (Kiilavuori et al. 2021, 2022; Linnunsalo et al. 2023). After the manipulation check, the experimenter debriefed and rewarded the participant.

#### 3.1.3 Data Acquisition and Analysis

Physiological signals were measured, post‐processed, and statistically analyzed as described in Experiment 1. Table 1 summarizes the proportions of rejected trials and the resulting numbers of excluded participants for each psychophysiological measure.

**TABLE 1:** The proportion of rejected trials and number of excluded participants for each psychophysiological measure.

Psychophysiological measure	Rejected trials	Excluded participants
Skin conductance	11.82%	12
Zygomaticus muscle activity	2.53%	1
Corrugator muscle activity	3.32%	1
Heart rate deceleration	6.17%	6

*Note:* A participant was excluded from analyses if they had more than three rejected trials in any stimulus condition.

### 3.2 Results

#### 3.2.1 Manipulation Check

All participants reported observing instances of eye contact and instances of no eye contact between the human and NAO. In addition, 46.9% of the participants also attributed affective connotations to the gaze conditions. Qualitatively, participants' descriptions of affective connotations were similar to those in Experiment 1, that is, participants seemed to have more unpleasant associations with non‐eye contact conditions (averted gaze, attempted eye contact, and avoided eye contact) compared to vicarious eye contact. Quantitatively, the likelihood of attributing affective connotations to the vicarious gaze conditions did not differ between Experiments 1 and 2 (*z* = 0.87, *p* = 0.384).

#### 3.2.2 Skin Conductance Results

##### 3.2.2.1 Vicarious Standpoint

**FIGURE 9:**
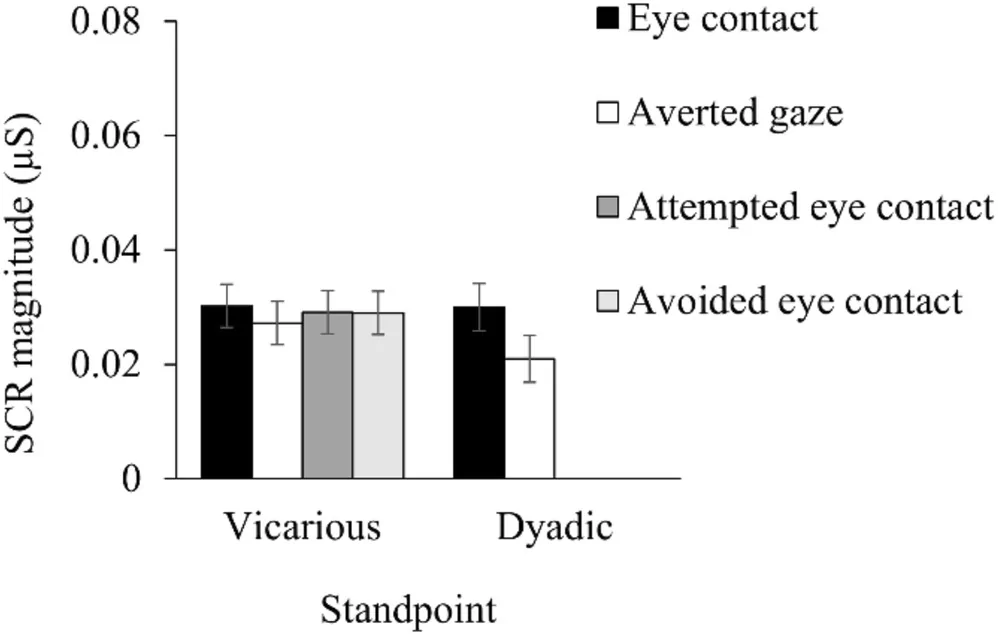
Mean skin conductance responses across gaze conditions, shown separately for the vicarious standpoint (participant viewing a person and a humanoid robot) and the dyadic standpoint (participant viewing a humanoid robot). Error bars represent the standard error. Response values were log10‐transformed.

The analysis showed no statistically significant main effect of gaze (*F*_(3, 2051.1)_ = 0.20, *p* = 0.893). Figure 9 illustrates the SCR results.

*Dyadic standpoint*. The results showed the main effect of gaze (*F*_(1, 994.18)_ = 5.97, *p* = 0.015), indexing more intense SCRs to eye contact (*M* = 0.030 μS, SEM = 0.0042) than averted gaze (*M* = 0.021 μS, SEM = 0.0041, *d*_z_ = 0.243).

#### 3.2.3 Facial Electromyography Results

##### 3.2.3.1 Vicarious Standpoint

**FIGURE 10:**
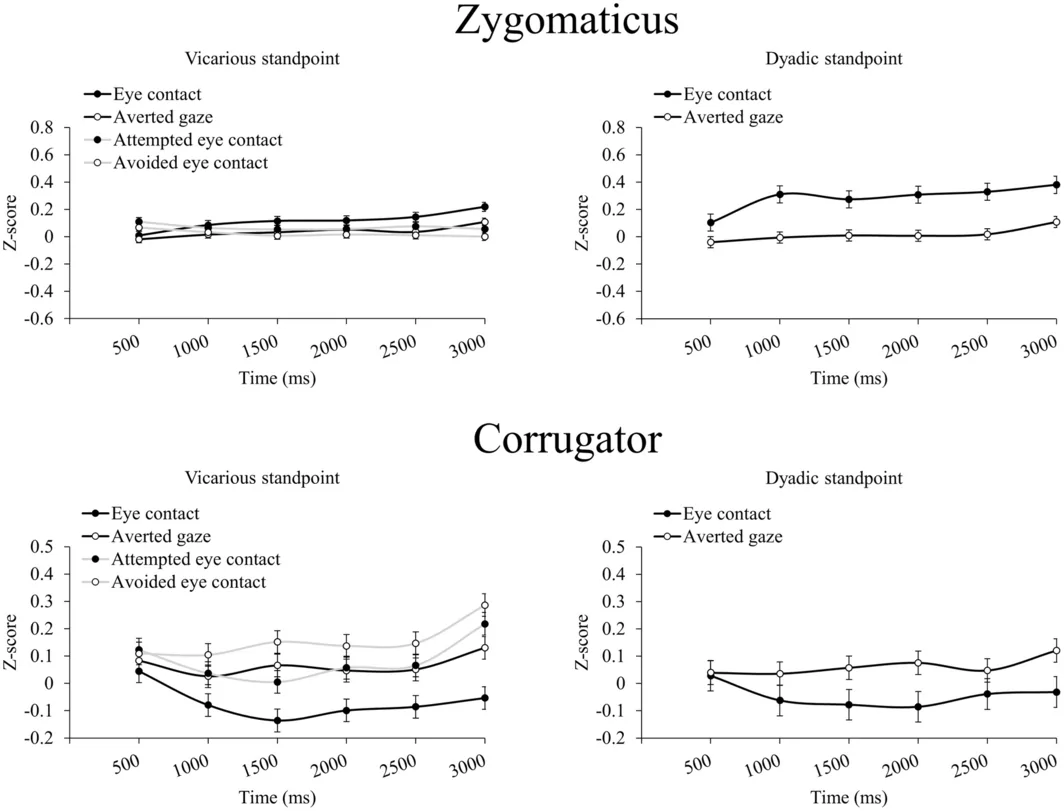
Mean zygomaticus and corrugator activity changes across gaze conditions, presented separately for the vicarious standpoint (participant viewing a person and a humanoid robot) and the dyadic standpoint (participant viewing a humanoid robot). Error bars represent the standard error. Response values were standardized.

For zygomaticus, the main effect of gaze was statistically significant (*F*_(3, 111.1)_ = 4.31, *p* = 0.006), suggesting that muscle activity increased more in response to eye contact (*M* = 0.12, SEM = 0.025) than averted gaze (*M* = 0.038, SEM = 0.016, *t*_(111)_ = 2.87, *p* = 0.005, *d*_z_ = 0.271), attempted eye contact (*M* = 0.070, SEM = 0.019, *t*_(111)_ = 1.98, *p* = 0.050, *d*_z_ = 0.187), and avoided eye contact (*M* = 0.020, SEM = 0.016, *t*_(111)_ = 3.48, *p* < 0.001, *d*_z_ = 0.329). The main effect of time was not statistically significant (*p* = 0.060), but there was a statistically significant Gaze × Time interaction (*F*_(15, 15,221.9)_ = 3.64, *p* < 0.001). The interaction suggested an increase in muscle activity as a function of time in response to eye contact (*F*_(5, 3795.6)_ = 7.93, *p* < 0.001) and averted gaze (*F*_(5, 3781.3)_ = 3.06, *p* = 0.009), but not in response to avoided eye contact and attempted eye contact (both *p*s ≥ 293). For corrugator, the main effect of gaze was statistically significant (*F*_(3, 15,600)_ = 46.41, *p* < 0.001). Compared to eye contact (*M* = −0.068, SEM = 0.028), muscle activity increased more in response to averted gaze (*M* = 0.067, SEM = 0.028, *t*_(15,600)_ = 6.94, *p* < 0.001, *d*_z_ = 0.656), attempted eye contact (*M* = 0.084, SEM = 0.028, *t*_(15,599)_ = 7.84, *p* < 0.001, *d*_z_ = 0.740), and avoided eye contact (*M* = 0.16, SEM = 0.028, *t*_(15,600)_ = 11.54, *p* < 0.001, *d*_z_ = 1.090). There was also a main effect of time (*F*_(5, 15,597)_ = 8.42, *p* < 0.001), suggesting an increase in muscle activity as a function of time. Finally, there was a statistically significant Gaze × Time interaction (*F*_(15, 15,597)_ = 1.71, *p* = 0.042), suggesting a change in muscle activity over time in response to eye contact, avoided eye contact, and attempted eye contact, but not in response to averted gaze. The results are illustrated in Figure 10.

##### 3.2.3.2 Dyadic Standpoint

For zygomaticus, the main effect of gaze was statistically significant (*F*_(1, 111.0)_ = 23.52, *p* < 0.001), indicating greater muscle activity increase in response to eye contact (*M* = 0.28, SEM = 0.053) than averted gaze (*M* = 0.016, SEM = 0.025, *d*_z_ = 0.458). Also, the main effect of time was statistically significant (*F*_(5, 7704.4)_ = 7.17, *p* < 0.001), indicating an increase in muscle activity over time. The interaction between gaze and time was not statistically significant (*p* = 0.167). For corrugator, there was also a main effect of gaze (*F*_(1, 109.3)_ = 4.82, *p* = 0.030), indicating that muscle activity increased more in response to averted gaze (*M* = 0.063, SEM = 0.031) compared to eye contact (*M* = −0.045, SEM = 0.048, *d*_z_ = 0.207). The main effect of time and the Gaze × Time interaction were not statistically significant (both *p*s ≥ 0.200).

##### 3.2.3.3 Comparison Between the Vicarious and Dyadic Standpoints

For zygomaticus, the main effect of gaze was statistically significant (*F*_(1, 111.4)_ = 26.63, *p* < 0.001); the muscle activity increase was greater in response to eye contact (*M* = 0.20, SEM = 0.034) than averted gaze (*M* = 0.030, SEM = 0.015, *d*_z_ = 0.488). Also, the main effect of standpoint was statistically significant (*F*_(1, 111.3)_ = 6.56, *p* = 0.012), indicating that the muscle activity increase was greater in dyadic (*M* = 0.15, SEM = 0.031) versus vicarious standpoint (*M* = 0.079, SEM = 0.017, *d*_z_ = 0.242). Finally, the interaction between gaze and standpoint was also statistically significant (*F*_(1, 111.9)_ = 13.30, *p* < 0.001). Pairwise comparisons suggested that the zygomaticus muscle activity was greater in response to dyadic eye contact (*M* = 0.28, SEM = 0.053) compared to vicarious eye contact (*M* = 0.11, SEM = 0.025, *t*_(111)_ = 3.56, *p* < 0.001, *d*_z_ = 0.336). The responses did not differ between dyadic and vicarious averted gaze (*p* = 0.310). For corrugator, the main effect of gaze was statistically significant (*F*_(1, 114.4)_ = 18.30, *p* < 0.001), indicating greater responses to averted gaze (*M* = 0.083, SEM = 0.024) compared to eye contact (*M* = −0.055, SEM = 0.034, *d*_z_ = 0.404). The main effect of standpoint and the interaction between gaze and standpoint were not statistically significant (both *p*s ≥ 0.302).

#### 3.2.4 Heart Rate Deceleration Results

##### 3.2.4.1 Vicarious Standpoint

**FIGURE 11:**
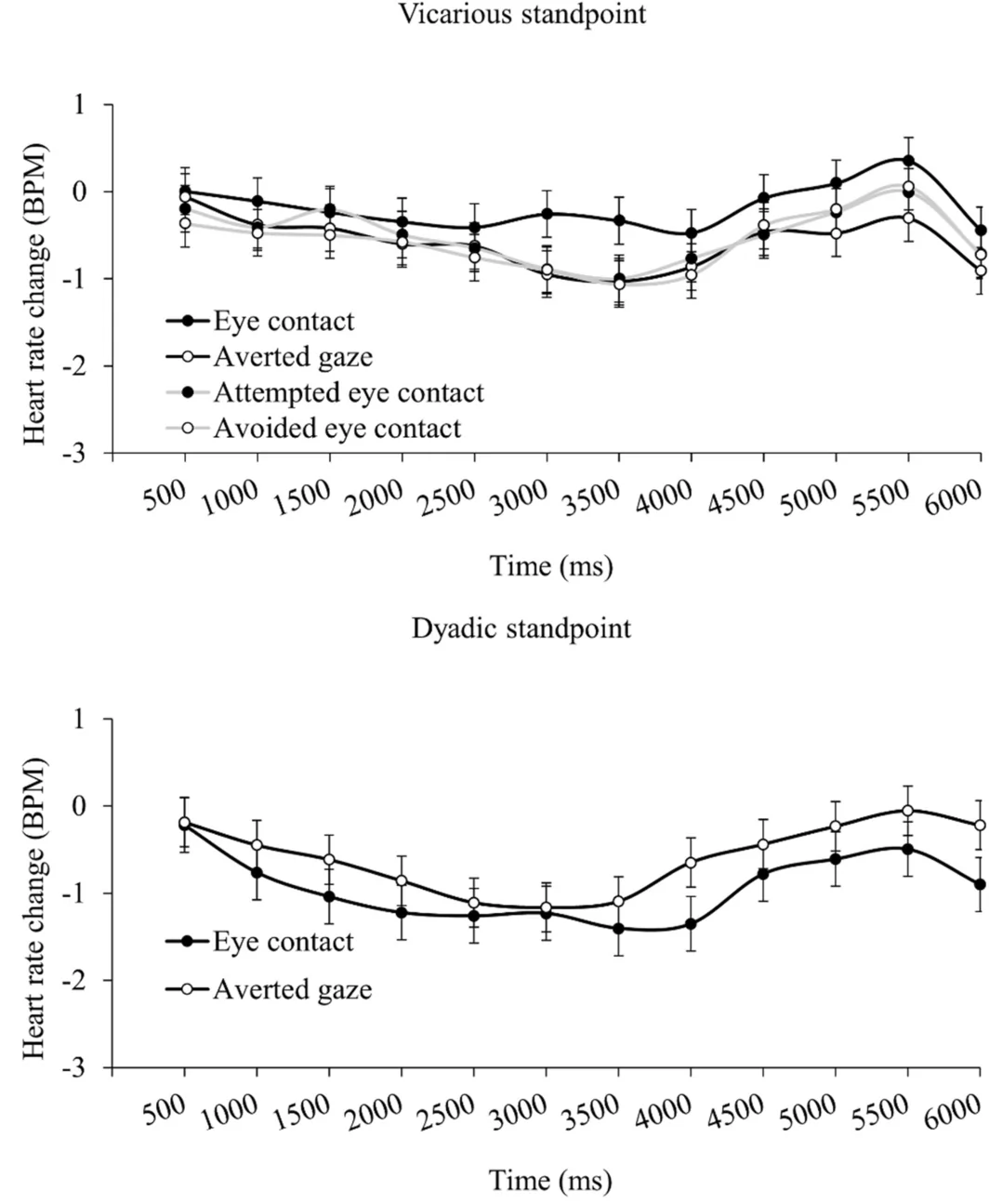
Mean heart rate changes across gaze conditions, shown separately for the vicarious standpoint (participant viewing a person and a humanoid robot) and the dyadic standpoint (participant viewing a humanoid robot). Error bars represent the standard error.

The results showed the main effect of gaze (*F*_(3, 30,308)_ = 7.16, *p* < 0.001). Strikingly, heart rate deceleration was *less* intense in response to eye contact (*M* = −0.19 BPM, SEM = 0.13), as compared to averted gaze (*M* = −0.59 BPM, SEM = 0.13, *t*_(30,313)_ = 4.07, *p* < 0.001, *d*_z_ = 0.393), attempted eye contact (*M* = −0.51 BPM, SEM = 0.13, *t*_(30,313)_ = 3.20, *p* = 0.001, *d*_z_ = 0.309), or avoided eye contact (*M* = −0.57 BPM, SEM = 0.13, *t*_(30,313)_ = 3.88, *p* < 0.001, *d*_z_ = 0.375). There was also a main effect of time (*F*_(11, 30,302)_ = 5.20, *p* < 0.001) demonstrating heart rate deceleration. The Time × Gaze interaction was not statistically significant (*p* > 0.999). The HRDR results are illustrated in Figure 11.

##### 3.2.4.2 Dyadic Standpoint

The main effect of gaze and the Gaze × Time interaction were statistically nonsignificant (both *p*s ≥ 0.189). A statistically significant main effect of time (*F*_(11, 15,112.2)_ = 4.94, *p* < 0.001) reflected heart rate deceleration.

### 3.3 Discussion

Contrary to our expectations, in the vicarious block, eye contact between a human and NAO did not elicit greater SCRs than non‐eye contact conditions. Thus, unlike during vicarious human–human eye contact, vicarious human–robot eye contact did not increase observers' affective arousal. On the other hand, vicarious human–robot eye contact did influence participants' fEMG responses, with increased zygomaticus activity and decreased corrugator activity relative to non‐eye contact conditions. Finally, although HRDRs differed between vicarious eye contact and non‐eye contact conditions, contrary to our expectations, they indicated weaker attention orienting during vicarious eye contact compared to non‐eye contact conditions.

For the dyadic block, we replicated the previous findings by Kiilavuori et al. (2021, 2022), showing that dyadic eye contact with a humanoid robot elicited greater SCRs as well as increased zygomaticus activity and decreased corrugator activity. However, contrary to previous findings (Kiilavuori et al. 2021), HRDRs did not differ between NAO's direct and averted gaze. We propose the same explanation for this null finding as in Experiment 1, namely, that the responses may have been attenuated by prior exposure to the same stimulus model in the vicarious block before the dyadic block.

## 4 General Discussion

Studies have consistently shown that direct gaze from another agent—human or humanoid robot—toward the observer (i.e., dyadic eye contact) elicits physiological responses indicating greater affective arousal, more positive affective valence, and enhanced attention orienting compared to averted gaze (J. K. Hietanen et al. 2008, 2018; Jarick and Bencic 2019; Kiilavuori et al. 2021, 2022; Myllyneva and Hietanen 2015; Nichols and Champness 1971; Prinsen and Alaerts 2019). In the present study, we investigated whether these affect‐ and attention‐related responses also occur when observers witness eye contact between two other agents, a phenomenon we termed the *vicarious eye contact effect*. We measured participants' SCRs indexing affective arousal, fEMG responses from the zygomaticus and corrugator areas reflecting affective valence and affiliation, and HRDRs reflecting attention orienting while they viewed gaze interactions between two agents. In Experiment 1, both agents were humans, whereas in Experiment 2, one was a human and the other was a humanoid robot. In Experiment 1, one human agent served as the person‐of‐interest, and participants were instructed to focus on that individual. In Experiment 2, the person‐of‐interest was always the human. Participants viewed four types of gaze interactions: eye contact between the agents and three non‐eye contact conditions (averted gaze, attempted eye contact, and avoided eye contact; see Section 2.1.2). We anticipated greater SCRs, increased zygomaticus activity, decreased corrugator activity, and more pronounced HRDRs during vicarious eye contact than during non‐eye contact conditions. We also measured the same psychophysiological responses to dyadic eye contact in which participants made direct eye contact with an agent (a human in Experiment 1 and a humanoid robot in Experiment 2) versus viewing that agent's averted gaze. This dyadic block allowed us to compare the eye contact effect (i.e., stronger responses to eye contact than to non‐eye contact conditions) between the vicarious and dyadic standpoints. We predicted that the responses would be stronger for dyadic than vicarious eye contact.

Observing eye contact between two humans increased observers' affective arousal compared to viewing non‐eye contact conditions. This vicarious eye contact effect is a novel contribution that extends current understanding of how eye contact influences affective arousal. The effect on SCRs is particularly noteworthy because previous work has shown that skin conductance is highly sensitive to the *psychological* components of eye contact, specifically, the experience of two minds engaging through mutual gaze. Prior studies indicate that the mere *visual* presentation of direct gaze does not elicit greater SCRs than averted gaze when participants believe that the confederate cannot see them because of an alleged one‐way window (Myllyneva and Hietanen 2015), or when direct gaze is presented in a static image or a pre‐recorded video (J. K. Hietanen et al. 2008; J. O. Hietanen et al. 2020; Prinsen and Alaerts 2019). These findings suggest that increased SCRs to eye contact reflect the experience of genuine social interaction, that is, a reciprocal awareness of being attended by another person (J. K. Hietanen et al. 2018), rather than simple perception of gaze direction. Thus, the present SCR findings suggest that this affective response may extend to third‐party observers witnessing eye contact between others. However, the effect of vicarious eye contact on affective arousal was weaker than that of dyadic eye contact, likely because of the greater self‐relevance of the latter.

Interestingly, there was no significant difference in affective arousal between vicarious eye contact and non‐eye contact conditions when the observed interaction involved a human and a humanoid robot. Previous research, as well as the dyadic block of the present study, has shown that dyadic eye contact with a humanoid robot elicits greater affective arousal than viewing its averted gaze (Kiilavuori et al. 2021, 2022). However, these studies also indicate that the effect of dyadic eye contact on affective arousal is generally weaker in human–robot interaction than in human–human interaction. This difference likely relates to the fact that people often attribute less mind to robots than to humans (Levin et al. 2008; Z. Li et al. 2022; Marchesi et al. 2019). Given that vicarious human–human eye contact elicited lower affective arousal than dyadic eye contact in the present study, and that previous studies have shown lower affective arousal in response to dyadic eye contact with a humanoid robot than with a human (Kiilavuori et al. 2021, 2022), it is plausible that these two attenuating factors—the vicarious standpoint and the involvement of a humanoid robot—jointly reduced the affective significance of vicarious human–robot eye contact. Consequently, the observed human–robot eye contact may not have engaged the sympathetic arousal system any more than non‐eye contact conditions.

The fEMG responses to vicarious eye contact versus non‐eye contact conditions did not differ during interaction between two humans. As argued earlier, this pattern may reflect the ambiguous affective valence of the observed interaction. It is possible that, for some participants, the interaction appeared affectively positive despite the absence of verbal or non‐verbal interaction cues from the confederates other than eye contact, whereas others may have perceived it less positively or even negatively. Interestingly, however, vicarious eye contact between a human and NAO increased zygomaticus activity and decreased corrugator activity relative to non‐eye contact conditions. At first glance, this finding may appear surprising given our assumption that gaze‐related effects would be stronger in human–human interaction than in human–robot interaction. One possible explanation lies in the lower degree of mind attribution to humanoid robots relative to humans (Levin et al. 2008; Z. Li et al. 2022; Marchesi et al. 2019). Because NAO is often perceived as a less intentional, more object‐like social agent, participants may have interpreted the human–robot gaze interaction as more straightforward and affectively unambiguous than an interaction between two humans. This reduced ambiguity could have facilitated clearer affiliative responses to vicarious eye contact, resulting in increased zygomaticus and decreased corrugator activity compared to the corresponding non‐eye contact conditions.

Contrary to our expectations, observed eye contact between two agents did not result in greater HRDRs than the other gaze directions. This finding applied to both human–human and human–robot conditions and, therefore, suggests that vicarious eye contact does not capture an observer's attention. Previous research, along with the present results, has demonstrated that in dyadic gaze interactions, direct gaze elicits greater HRDRs than averted gaze (Akechi et al. 2013; Myllyneva and Hietanen 2015). The ability of direct gaze to capture attention has also been demonstrated in several behavioral studies, where faces with direct gaze were detected more quickly than those with averted gaze (Conty et al. 2006; Senju et al. 2005; von Grünau and Anston 1995). Thus, the present results suggest that observed eye contact, whether between two humans or between a human and a humanoid robot, lacks the attentional saliency of dyadic eye contact. Instead, in vicarious human–human interaction, viewing a person attempting to engage in eye contact but receiving no response elicited greater HRDRs than the other gaze conditions. As discussed earlier, the attempted eye contact condition may have been interpreted as indicating that the POI was being socially excluded by another individual, resulting in stronger attentional orienting. However, given the limited subjective data available, this interpretation remains speculative.

Interestingly, in vicarious human–robot interaction, viewing any non‐eye contact gaze condition (i.e., both parties looking away, or one looking at the other while the other looks away) elicited greater HRDRs than eye contact. A speculative explanation for this finding is that people may be most familiar with depictions of a humanoid robot and a human gazing at each other, as this gaze configuration is commonly used to represent human–robot interaction in contexts such as news media, advertising, and art. Because people may still have limited first‐hand experience with humanoid robots, gaze configurations that deviate from this familiar eye contact scenario may be perceived as novel, thereby attracting attention and eliciting greater HRDRs. However, the psychophysiological data alone do not allow definite conclusions about the mechanisms underlying this finding.

The present study has some limitations that are important to consider. First, although we found a novel effect of vicarious eye contact on psychophysiological responses, we have limited subjective data to complement our understanding of participants' affective experiences. Several previous studies investigating affective responses to dyadic eye contact have used subjective measures, such as the Self‐Assessment Manikin (see Bradley and Lang 1994), alongside psychophysiological measures (Akechi et al. 2013; J. K. Hietanen et al. 2008, 2018; Kiilavuori et al. 2021). While it is not uncommon for subjective results to differ from those obtained through more objective methods (e.g., Evans 2008; Hofmann et al. 2005; Kiilavuori et al. 2021), self‐reported affective responses remain an important source of information. In the present study, we did not specifically ask participants about their subjective affective responses to vicarious gaze conditions. However, some participants reported affective impressions of the gaze conditions during the manipulation check. Unfortunately, this measure was too vague to support strong conclusions about the observed findings.

Related to the limitation discussed above, the present experimental paradigm makes it difficult to draw strong conclusions about the mechanisms underlying the vicarious eye contact effect. Specifically, it remains unclear whether participants' psychophysiological responses were primarily driven by adopting the POI's perspective and vicariously experiencing the POI's affective responses to dyadic eye contact, or whether they instead reflected participants' own affective reactions to observing eye contact between other individuals. We consider the former explanation more plausible, given that participants were explicitly instructed to consider how the POI might be feeling during the gaze trials. Moreover, participants' responses to the manipulation check generally reflected alter‐centric interpretations of the gaze conditions; they described the POI's gaze in relation to the other person/robot rather than to themselves. Nevertheless, further research is needed to clarify the mechanisms underlying the vicarious eye contact effect.

Finally, in Experiment 2, we did not ask participants about their previous experiences with humanoid robots (e.g., number of previous interactions or interest in robot development). Such information would have helped interpret the HRDR results, as it could have allowed us to investigate whether attention orienting toward vicarious human–robot eye contact was influenced by prior experience with human–robot interaction. A previous study measuring psychophysiological responses to dyadic eye contact with a humanoid robot collected data on participants' prior experiences with humanoid robots but found no correlations between these factors (Kiilavuori et al. 2021). However, these findings do not rule out the possibility that prior experiences with humanoid robots influenced their psychophysiological responses to vicarious human–robot eye contact.

The present study highlights the value of using both natural and artificial agents as stimuli when investigating social cognition. Including artificial agents is important for several reasons. First, artificial agents such as humanoid robots are becoming more common in social environments, and humans are expected to interact with them socially. Research on social cognition in human–robot interaction can help developers design robots that behave more naturally and integrate smoothly into everyday environments. Second, people often attribute mind to artificial agents (H. M. Gray et al. 2007; Huebner 2010; Ward et al. 2013), although typically less than to humans (Levin et al. 2008; Marchesi et al. 2019). Using artificial agents can therefore provide unique insights into how mind attribution shapes social‐cognitive processes. For example, the experience of being seen by another mind is considered essential for many affect‐ and attention‐related effects of eye contact (Conty et al. 2016; J. K. Hietanen 2018; J. K. Hietanen et al. 2018; Myllyneva and Hietanen 2015). Yet manipulating mind attribution toward humans is difficult and impractical, whereas previous work has successfully manipulated the degree of mind attributed to artificial agents to examine its influence on social cognition (Caruana et al. 2017; Linnunsalo et al. 2023; Wykowska et al. 2014). Therefore, incorporating artificial agents alongside humans can meaningfully complement research on human social cognition.

In conclusion, the present study showed that vicarious eye contact elicited psychophysiological responses related to affective arousal and valence. Specifically, observing eye contact between two humans elicited greater skin conductance responses than viewing non‐eye contact conditions, though participants' fEMG responses did not differ across these human–human gaze conditions. In human–robot interaction, the pattern differed: observing eye contact between a human and a humanoid robot did not increase affective arousal, but facial muscle activity suggested positive affective valence or an affiliative response. These findings provide novel insights into human reactions to eye contact, demonstrating that it can elicit affect‐related responses even when the observer is not directly involved. Further research is needed to better understand the effects of eye contact in multi‐person interactions involving both natural and artificial agents.

## Author Contributions

**Samuli Linnunsalo:** conceptualization, methodology, software, data curation, formal analysis, supervision, resources, validation, visualization, investigation, writing – original draft, writing – review and editing. **Jari K. Hietanen:** conceptualization, methodology, writing – review and editing, supervision, project administration, funding acquisition.

## Funding

This work was supported by Academy of Finland (330158).

## Conflicts of Interest

The authors declare no conflicts of interest.

## Data Availability

The study was preregistered prior to any data collection, and the preregistration is available at https://doi.org/10.17605/OSF.IO/Q7VHS. The research data and analysis code are available at https://osf.io/mpds7/overview?view_only=2490e4e0bac34fa0944be7b3a52e030b.
